# Interoceptive accuracy and its impact on neuronal responses to olfactory stimulation in the insular cortex

**DOI:** 10.1002/hbm.24985

**Published:** 2020-03-26

**Authors:** Carina J. Koeppel, Paul Ruser, Hagen Kitzler, Thomas Hummel, Ilona Croy

**Affiliations:** ^1^ Department of Psychotherapy and Psychosomatic Medicine TU Dresden Faculty of Medicine Carl Gustav Carus Dresden Germany; ^2^ Clinic for Diagnostic and Interventional Neuroradiology TU Dresden Faculty of Medicine Carl Gustav Carus Dresden Germany; ^3^ Smell & Taste Clinic, Department of Otorhinolaryngology TU Dresden Faculty of Medicine Carl Gustav Carus Dresden Germany

**Keywords:** exteroception, insular cortex, interoception, interoceptive accuracy, olfaction

## Abstract

The insular cortex plays a key role in the integration of multimodal information and in interoceptive and exteroceptive processing. For instance, neurons in the central dorsal insula that are active during interoceptive tasks, also show an adaptation to gustatory stimulation. We tested the link between interoception and exteroception for the olfactory system (i.e., the second domain of chemosensation). In a sample of 31 participants, olfactory function was assessed in a two dimensional approach while the Heartbeat Perception Task served as a measurement for cardiac interoceptive accuracy. Subsequent fMRI sessions were performed on a 3‐Tesla MR scanner containing 12–15 olfactory stimulation trials with a mildly pleasant food‐related odor (coffee). Persons scoring high in the cardiac interoceptive accuracy task presented stronger smelling abilities as well as enhanced BOLD responses following olfactory stimulation. The olfactory stimulation triggered enhanced insular activation patterns in the central dorsal insular cortex. Consistent with prior findings on the coherence of gustatory and interoceptive processing in the central dorsal insula, these results base the insula as a common region for the integration of interoception and exteroception. We propose an explanatory model of how exteroception triggers the integration of intero‐ and exteroceptive sensations in the central dorsal insular cortex.

## Graphical abstract

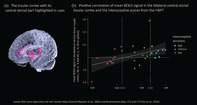

The integration of intero‐ and exteroceptive input is crucial for detecting the salience of surrounding stimuli. Participants with high cardiac interoceptive accuracy present stronger central‐dorsal insular activation patterns during olfactory stimulation. An explanatory model is proposed focusing on an exteroceptive trigger for the multimodal integration in the central‐dorsal insula.

## 1 INTRODUCTION

In human perception, a division is often made between perception of the external environment, and perception of the physiological condition. The former, termed exteroception, is the perception of surrounding stimuli through the classical sensory organs. The latter, termed interoception, is the perception of internal bodily states, through signals coming from within the body. For instance, bladder distension, heart palpitations, or the feeling of fullness are defined as interoceptive stimuli (Craig, 2002; Tan et al., 2018). It is only through the integration of exteroceptive and interoceptive stimuli, that an organism can experience itself as an integrated entity (Damasio, 1999). Intero‐ and exteroceptive stimuli are constantly updated, and disturbances leading to an imbalance in homeostasis are immediately processed and recognized, with balance restored by the initiation of adapting countermeasures (Craig, 2007). As the insular cortex is a key structure in monitoring the relevance of incoming sensory information and maintaining homeostasis (Menon & Uddin, 2010), it is considered as the integration center of intero‐ and exteroception (Craig, 2009).

Interoceptive sensations reach the posterior insular cortex by two major tracks—First, through the laminaI spinothalamic pathway, and second, through afferents relaying input from cranial nerves IX and X to the solitary nucleus (Craig, 2002; van der Kooy, Koda, McGinty, Gerfen, & Bloom, 1984). Both sympathetic and parasympathetic afferent pathways connect to the ventromedial posterior nuclei thalami either directly, or by passing the parabrachial nucleus first. From there, the peripheral‐sensory information is forwarded along the posterior insula to the mid and anterior insula (Craig, 2002).

Exteroceptive sensations like visual, auditory, gustatory, or somatosensory input reach higher processing and association centers by the lateral geniculate body, the medial geniculate body or the ventral posteromedial nucleus (VPM) of the thalamus (Lambert, Simon, Colman, & Barrick, 2017). The olfactory input, however, is directly mediated to the insular cortex, without passing the thalamus first (Gottfried, 2006). Both gustatory and olfactory input converge in the anterior‐dorsal insula—a subdivision of the insula, which is specialized in chemosensory processing (Kelly et al., 2012; Kurth, Zilles, Fox, Laird, & Eickhoff, 2010).

Consistent with its central location, the insula serves as a meeting and distribution point—integrating information from the autonomic nervous system and guiding subsequent higher cognitive responses (Uddin, 2015). The insula stands out by its broad cortico‐cortical as well as cortico‐subcortical network. In particular, the connections from the insular to the cingulate cortex seem to be intensified in a specific order: the anterior insula is more linked to the anterior cingulate cortex while the posterior insula is more linked to the posterior cingulate cortex (Ghaziri et al., 2017). The connections to the cingulate cortex enable forwarding information to initiate behavioral responses. Further, connections to the amygdala, the nucleus accumbens and the thalamus allow for sensory processing, reward, and emotional responses (Ghaziri et al., 2018).

The convergence of exteroception and interoception in the insula enables salience detection (Menon & Uddin, 2010). Consequently, the perception of internal stimuli is crucial for the organism to interpret the salience of surrounding external stimuli. For instance, the warm smell of freshly cooked bread is generally more inviting for people with an empty stomach and low blood sugar levels, than sated individuals (with high blood sugar levels).

In the long run, interoceptive abilities serve the purpose to selectively attend to those external stimuli which are important to keep in balance according to the current interoceptive demands. The insula's specialized function in sensory integration is supported anatomically by convergence of interoceptive and exteroceptive information along posterior to anterior pathways in the insula. Specifically, interoceptive information is processed in more posterior and middle parts and integrated with exteroceptive input (e.g., from olfaction) in middle to anterior parts. Finally, salience detection—resulting from combined information—is located in anterior parts of the insula (Craig, 2011; Kelly et al., 2012; Kurth et al., 2010).

We assume that intero‐ and exteroceptive abilities impact each other and that the interoceptive ability shapes the ability to attend to external stimuli in the long run. For instance, a person who is very prone to perceive her own heartbeat may be more aware of potentially dangerous environmental situations, and identify such situations more frequently than a person with lower heartbeat sensitivity.

So far, studies focusing on both, intero‐ and exteroceptive abilities in the same study sample are rare. For olfaction and interoception we only know of the one done be Krajnik, Kollndorfer, Notter, Mueller, and Schopf (2015). In an interoceptive‐attention paradigm, Stern et al. (2017) examined the performance of healthy individuals, in rating the brightness/blinking rate of words while monitoring their skin temperature/heartbeat (Stern et al., 2017). The researchers found no interaction between visual perceptive abilities and interoceptive abilities.

In contrast, studies in chemosensory domains, show different results. For gustation, a recent fMRI study found a specific group of neurons in the central dorsal insula that were sensitive to both gustatory and interoceptive tasks (Avery et al., 2017). The central dorsal insula further presents a chemosensory overlap of gustatory and olfactory sensations reflected in clinical responses to electrical stimulation of this brain area (Mazzola et al., 2017). Stimulation of the central dorsal insula also evoked oral somatosensation, leading to the hypothesis of the central dorsal insula as an integrated oral sensory region (Mazzola et al., 2017). For olfaction, it was found that dysosmic participants had lower cardiac interoceptive accuracy compared to normosmics, as reflected in reduced scores in the mental tracking method. These attenuated scores for dysosmics correlated with the duration of reduced olfactory functioning (Krajnik et al., 2015). The findings suggest a situational interaction of intero‐ and exteroceptive stimulus processing which may extend to long‐term consequences in the interplay of intero‐ and exteroception.

Guided by prior findings on the relation of olfactory threshold and interoceptive awareness by Krajnik et al. (2015), we assume that the relation is bidirectional and hypothesize that people with high cardiac interoceptive accuracy also score high in all subtestings of olfactory function (olfactory discrimination, olfactory identification, olfactory threshold). Further, we assume that this relation of interoception and exteroception is reflected in enhanced neuronal responses to olfactory stimuli in the insular cortex in people with high interoceptive abilities.

## 2 METHODS

### 2.1 Study sample

#### 2.1.1 Ethic statement

The study followed the declaration of Helsinki on Biomedical Research involving human subjects and was approved by the local ethics committee of the Technical University Dresden. All participants were informed about the study paradigm and data security, which was documented by written consent.

#### 2.1.2 Participants

In total 36 participants (24 women, 12 men, aged between 18 and 36 years, mean: 24, 2 years, *SD*: ±4.1) were included in the study. Exclusion criteria were (a) mental disorders as screened by the Patient Health Questionnaire (Kroenke, Spitzer, Williams, & Lowe, 2010), as those may impact interoceptive abilities; (b) olfactory dysfunction reflected in a combined olfactory threshold‐discrimination‐identification score below 30 (Hummel, Kobal, Gudziol, & Mackay‐Sim, 2007), as we aimed to draw conclusions for normosmic individuals; and (c) general diseases presenting an impact on smelling abilities such as diabetes mellitus, renal insufficiency, and acute or chronic sinusitis. Due to time and MRI capacity limitations, only the first 33 participants were invited to participate in the olfactory fMRI experiment (11 participants on each testing day). Due to drop outs, the remaining fMRI group consisted of 31 participants (19 women, 12 men, aged between 18 and 36 years, mean age 24, 3 years ± 3.71 *SD*). All subjects were right‐handed.

### 2.2 Procedure

The study was embedded in a larger project themed on the hedonic value of odors. As individual odors might evoke different brain signatures, we based our hypothesis—driven analysis on the group‐neutral odor, which was identical for all participants. This project included an extensive pre‐testing to determine subjective odor preference before the fMRI measurements. There were three functional runs in the scanner per participant—Presentation of the individually most‐preferred odor, presentation of the individually least‐preferred odor, and presentation of a neutral‐odor, which was the same for each participant. The present analysis is based on the neutral odor condition (Coffee, medium valence = 3.31 + −1.22*SD*, Scales 1 (unpleasant) to 5 (pleasant), brand: fragrance resource, Hamburg, Germany). In line with the focus of the present study and for sake of brevity, description of the pretesting procedure and the two other functional runs focusing on the hedonic valence is omitted.

#### 2.2.1 Psychophysical testing of chemosensory function

Smelling abilities were tested by application of the “Sniffin' Sticks” Battery (Burghart GmbH, Wedel, Germany), ensuring olfactory functioning in a two dimensional approach. Firstly, the olfactory threshold testing reflects basal peripheral functioning—*do I smell anything?* Here, the participants were asked to identify the odor‐containing pen among the solvent‐containing pens in varying dilutions. Secondly, the discrimination and identification testings relate to higher cognitive processing of olfactory sensations*—what do I smell?* (Hedner, Larsson, Arnold, Zucco, & Hummel, 2010). For olfactory discrimination, the participants were asked to smell three odor‐containing pens and to name the pen that smells different to the other two (repeated 16 times). For olfactory identification, the participants smelled 16 commonly known odors and identified them by choosing from four pictures presenting possible odors. For more details, please see Hummel et al. (2007).

#### 2.2.2 Interoceptive accuracy

There is marked heterogeneity in the use of interoceptive awareness and its subconstructs in the literature. For example, interoceptive accuracy has been defined as both a dimension of interoception, relating to the “objective accuracy in detecting internal bodily sensations,” (Garfinkel, Seth, Barrett, Suzuki, & Critchley, 2015, p. 3, Table 1), and as a feature of interoceptive awareness describing the ability to “precisely and correctly monitor changes in internal body state,” (Khalsa et al., 2018, supplement, p. 4). For reasons of clarity, we use interoception as umbrella term for the interoception sciences in general, while interoceptive awareness is used as the umbrella term for interoceptive sensations that are consciously available. Thus, under this classification, cardiac interoceptive accuracy (i.e., an individual's correct perception of their heart rate) is a feature of interoceptive awareness and at the same time a part of interoception.

**Table 1:** Sample description. Thirty‐one participants took part in the fMRI study of which 19 were female and 12 were male

		Olfactory subtestings			Cardiac interoceptive accuracy	
	Age	Threshold	Discrimination	Identification	First block	Second block
Mean	24.3	11.0	12.2	13.2	0.713	0.767
Median	24.0	10.5	12	13	0.723	0.792
Standard deviation	3.71	1.95	1.49	1.21	0.170	0.157
Minimum	18.0	7.50	9	11	0.371	0.351
Maximum	36.0	15.3	15	15	0.972	0.981

In this study, interoceptive accuracy was tested with the mental tracking method. We, therefore, used a modified version of the Heartbeat Perception Task (HBPT; Schandry, 1981), as described in another study examining the impact of olfactory dysfunction on interoceptive awareness (Krajnik et al., 2015). According to this restricted method of assessing interoceptive accuracy, we can only conclude for cardiac interoceptive accuracy. For this modified version, participants were asked to intrinsically feel and silently count their own heartbeats during three different counting phases of 30s, 20s, and 40s. At the same time, the study coordinator held onto the wrist of each participant and counted their heartbeats by feeling their pulse. The subjectively estimated values were compared to the objectively observed ones, and an index was calculated according to the following formula $\frac{1}{3}\sum 1-\frac{\text{observed}-\text{counted}}{\text{observed}}$ (Schandry, 1981; Stern et al., 2017).

**Figure 1:**
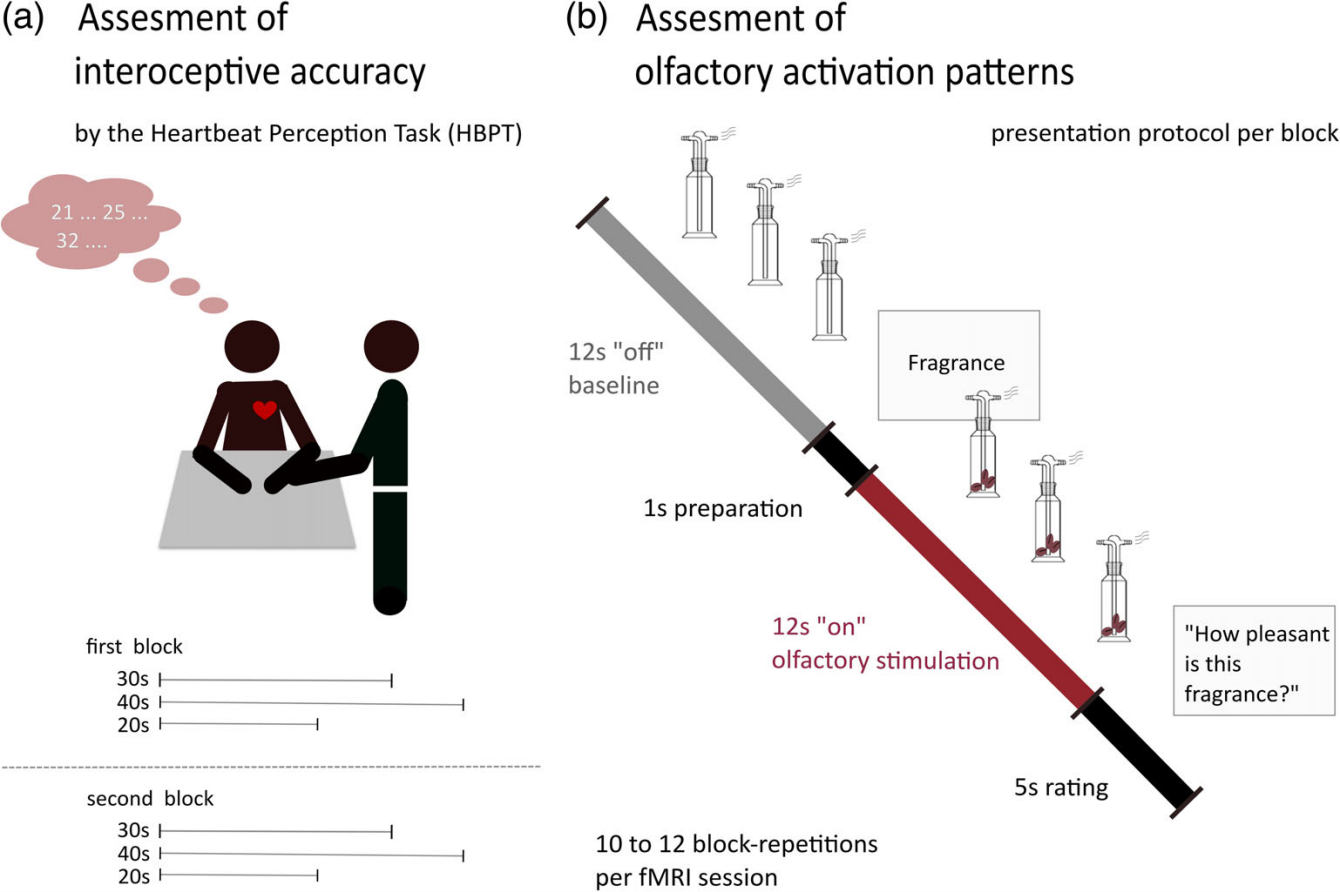
Study protocol. (Left panel) Every participant took part in a modified version of the Heartbeat Perception Task (HBPT). Individuals were instructed to silently count their heartbeats during three runs (30s, 40s, and 20s). These counted scores were compared to the ones observed by the study coordinator holding on to the participant's wrist (A. radialis pulse). (Right panel) Basic presentation protocol per block. Every session consisted of 10–12 on–off‐blocks. The duration per block is dependent on the individual respiration cycle, with approx. 12 s of odor presentation per olfactory stimulation (ON) and 12 s of air presentation per baseline (OFF). After each baseline, the word “fragrance” was presented on a monitor for 1 s. The following exhalation triggered the presentation of the next odor

The HBPT or mental tracking method was performed six times per person, divided into two blocks with three runs per block (Figure 1). The cardiac interoceptive accuracy was tested across two blocks to see if the measurements show to be reliable over time. Due to test effects, we based all statistical analyses on the mean cardiac interoceptive accuracy scores of the “naive” first block.

#### 2.2.3 fMRI—Data acquisition

Data collection was performed with an eight‐channel head‐coil on a 3‐Tesla MR scanner (Trio; Siemens Medical, Erlangen, Germany) at the University Hospital Dresden. Each participant took part in a functional session with a coffee odor, consisting of 120 volumes with 36 axial slices (slice‐thickness 3; imaging matrix 384 × 384; TR 3,000 ms; TE 40 ms; FA 90°; voxel size 3 × 3 × 3 mm; no interslice gap). These slices were transversally acquired with a 2D GE echo‐planar imaging sequence. In addition, high resolution T1‐weighted image were conducted for anatomical mapping and exclusion of potential brain pathology in participants (3D IR/GR sequence: imaging matrix 384 × 384; TR 1,820 ms/TE 3,24 ms, FA 15; voxel size 1 × 1 × 1 mm).

The coffee‐fragrance was presented using a respiration triggered olfactometer (Sezille et al., 2013). Two 10‐m long, 4‐mm diameter polyurethane tubes from the olfactometer (placed outside of the scanning room) reached a mixing head that was located next to the participant. From this mixing head, two 4‐mm diameter polyurethane tubes led the odor into the participant's nostrils at a constant airflow of 1 L/min. The total duration of the session was set to 6 min, consisting of 10–12 on–off‐stimulation periods, with 12 s odor presentation followed by 12 s baseline (airflow only). During the sessions, participants were instructed to keep their eyes open and to focus on a screen, where the instructions were constantly displayed. After each baseline, the monitor presented the word “fragrance” for 1 s and thereafter the next exhalation triggered the presentation of the next olfactory stimulation. Due to the exhalation triggered onset, the interstimulus period varied depending on the individual respiration cycle. After each odor presentation, the monitor presented the question “How pleasant was this fragrance?” for 5 s. Participants were instructed to make the pleasantness‐rating using a five‐finger keyboard, where the first finger (thumb) indicated a very unpleasant percept, the fifth finger (little finger) indicated a very pleasant percept, and the other fingers indicating consecutive values in‐between (Figure 1).

### 2.3 Statistical analysis

#### 2.3.1 Behavioral data

Statistical analysis was performed by the SPSS Statistics Software (IBM Corp. Released 2017. IBM SPSS Statistics for Windows, Version 25.0. Armonk, NY: IBM Corp.), graphics were created in R (R Core Team 2018. R: A language and environment for statistical computing) and tables with jamovi (jamovi project (2018). jamovi (Version 0.9) [Computer Software]. Retrieved from https://www.jamovi.org).

In SPSS, a linear regression analysis was performed for the calculated means of the first and the second cardiac interoceptive accuracy task block. Comparisons of the means were done by a paired *t* test. The cardiac interoceptive accuracy scores were subcategorized according to “low” (score < 0.60), “medium” (0.60 < score < 0.85), and “high” (score > 0.85) cardiac interoceptive accuracy. The threshold score of 0.85 was adapted according to a prior study (Pollatos, Gramann, & Schandry, 2007), while the threshold score of 0.62 was determined by a median split of those individuals scoring below 0.85.

Multiple linear regression analysis was calculated with cardiac interoceptive accuracy as the dependent variable and the three olfactory subtestings as the independent variables (i.e., olfactory discrimination, olfactory identification, and olfactory threshold). Further linear regression analysis was conducted, by entering each olfactory subtesting separately as independent variables.

#### 2.3.2 fMRI analysis

For the fMRI analysis, a standard GLM approach was implemented in SPM12 (Statistical Parametric Mapping; Welcome Department of Imaging Neuroscience, in the Institute of Neurology at University College London [UCL], UK) and incorporated in Matlab (Matlab 9.1, The MathWorks IncS., Natick, MA). Preprocessing of the imaging data started with realignment and coregestering with fourth degree B‐spline; thereafter normalization was performed using the segmentation procedure implemented in SPM 12 with affine registration to the ICBM space template (MNI space), bias regularization of 0.0001, and spatial smoothing with a Gaussian kernel of 6 × 6 × 6 mm^3^ FWHM. Individual motions‐based noise was used as a regressor in the analysis, if movement during the runs was outside the tolerance range of <2 mm in translation and <1° in rotation. This was the case for seven participants. The first level statistical analysis was based on an individual ON–OFF‐Block design. Due to the long TR (3 s), the inert olfactory stimuli and the expiration triggered olfactometer, the OFF period was coded with a duration of three scans, while the ON period was coded with a duration of two scans.

**Figure 2:**
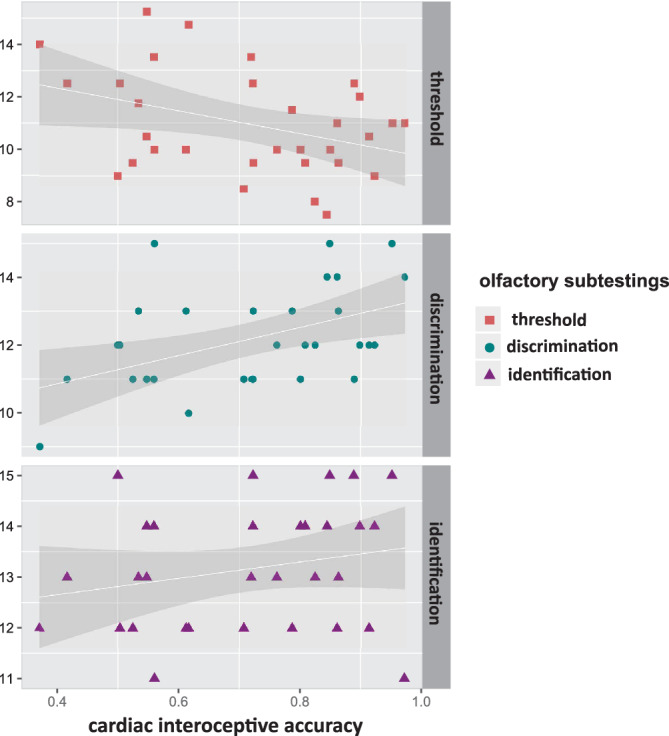
Linear regression analysis for all three olfactory subtestings measured in the Sniffin'Sticks method. (a) Olfactory discrimination and interoceptive accuracy (*R* = .475, *R* ^2^ = .226). (b) Olfactory identification and interoceptive accuracy (*R* = .225, *R* ^2^ = .051). (c) Olfactory threshold and interoceptive accuracy (*R* = .376, *R* ^2^ = .141). Overall, multiple regression analyses revealed an explained variance of 29% for the olfactory scoring

For the second level analysis, individual first level results were entered in a factorial design.

##### Olfactory activation

The odor versus baseline activation was inspected for the whole group of participants using a *t*‐test. Guided by prior findings on chemosensory activation patterns in the insula (Kurth et al., 2010), the family‐wise error (FWE) small volume correction was based on the left and right anterior dorsal insula for each of the regions of interest (ROIs) 167,168 taken from the brainnetome atlas (Fan et al., 2016). In order to compare the olfactory activation in the present study to prior findings on olfactory activation in the insular cortex, a 4‐mm sphere was created around the left and right insular activation peaks in the wfu pickatlas (Maldjian, Laurienti, Kraft, & Burdette, 2003). The spheres were visually compared to spheres created around the peak levels for the functional domains of chemosensory processing (MNI; right = 42, 13, −4; left = −30, 16, 5) as described in a meta‐analytic approach by Kurth et al. (2010). Whole‐brain activations of the odor versus baseline contrast are reported in the supplement (Table S1).

##### Covariate analysis

In the next step, the cardiac interoceptive accuracy scores were added as a covariate to the factorial design, in order to determine olfactory processing regions covarying with cardiac interoceptive accuracy. For the hypothesis driven focus on the central dorsal insula (Avery et al., 2017), the FWE‐small volume correction was based on the left and right central‐dorsal insula for each of the ROIs 171, 172, 173, 174 taken from the brainnetome atlas (Fan et al., 2016). Again, a 4‐mm sphere was created around the observed insular activation peaks in the wfu pickatlas (Maldjian et al., 2003). The spheres were visually compared to the spheres created around the peak levels for the functional domains of interoception (MNI; right = 41, 2, 3; left = −43, −3, 6) as described in a meta‐analytic approach by Kurth et al. (2010).

In order to visualize the individual patterns in relation to the interoceptive accuracy, the mean activation of the created 4‐mm spheres (around MNI peaks: right = 45, 2, −4; left = −45, 4, −4) was extracted per individual using MarsBar (Matthew Brett, Valabregue, & Poline, 2002). Whole‐brain activations of the covariate analysis are reported in the supplement (Table S2).

## 3 RESULTS

### 3.1 Behavioral measures

The participants presented a cardiac interoceptive accuracy score of 0.71 (±0.17 *SD*) in the first block and 0.76 (±0.16 *SD*) in the second block, with a large individual variation ranging from 0.35 to 0.98 (compare Table 1).

Cardiac interoceptive accuracy was significantly augmented in the second block (*T* = −2.618, *p* = .014). The correlation between both blocks was high (*R* = .756, *p* ≤ .001). In the first “naive” block, eight individuals could be classified as high scorers, 12 as medium scores, and nine as participants with low interoceptive ability. Cardiac interoceptive accuracy related significantly to the performance in the olfactory subtestings. The dimensions of olfactory discrimination, olfactory threshold and olfactory identification explained 29% of the variance of cardiac interoceptive accuracy (*R* = .538, *F* = 3.67, *p* = .024; Table 2 ).

**Table 2:** Regression analysis. Dependent variable: mean interoceptive accuracy, first block; *R* = .538, *R* ^2^ = .290

Variable	*B*	*SE B*	*β*	*t*	*p*
Threshold	−.015	.016	−.168	−.906	.373
Discrimination	.045	.021	.390	2.127	.043
Identification	.026	.023	.184	1.124	.271

Individual linear regression analysis performed for each of the olfactory subtestings revealed an explained variance of 22.6% by olfactory discrimination (*R* = .475, *p* = .007), 0.05% by olfactory identification (*R* = .225, *p* = .223) and 14.1% by olfactory threshold (*R* = .376, *p* = .037). While olfactory threshold related negativly, olfactory discrimination showed a positive relation (Figure 2).

**Figure 3:**
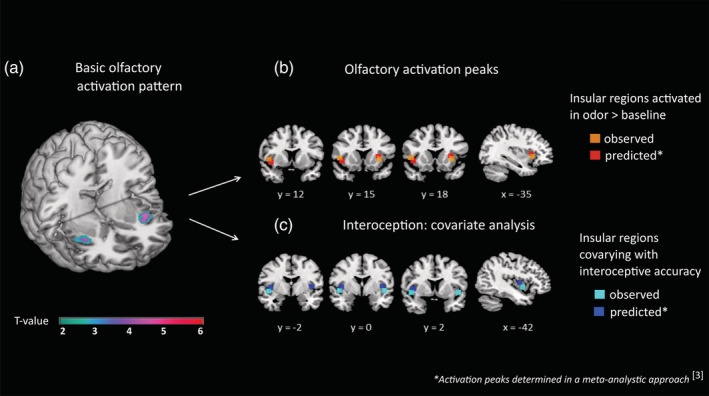
(a) Basic olfactory activation. FWE small volume correction was based on the left and right anterior dorsal insula for each of the ROIs 167,168 taken from the brainnetome atlas (Fan et al., 2016). (b) Olfactory activation peaks. Olfactory processing areas with activation peaks from the meta‐analysis shown in red, activation peaks of the present study shown in orange (right: *T* = 5.09, FWE_corr_ = .000; MNI: 37,18,4; left: *T* = 4.37, FWE_corr_ = .001; MNI: −33,18,0). (c) Interoception: Covariate analysis. Interoceptive processing areas with activation peaks from the meta‐analysis shown in blue and activation peaks from the present study (right: *T* = 3.71, FWE_corr_ ≤ .042; MNI: 45,2,−4; left: *T* = 3.51, FWE_corr_ ≤ .048; MNI: −45,4,−4) highlighted in cyan

### 3.2 fMRI measures

The olfactory stimulation led to a bilateral activation of the insula with insular activation peaks (right: *T* = 5.09, FWE_corr_ = .000; MNI: 37,18,4; left: *T* = 4.37, FWE_corr_ = .001; MNI: −33,18,0) located in the anterior dorsal insula in close proximity to the olfactory meta‐analytic activation peaks (Kurth et al., 2010, compare Figure 3).

A linear regression analysis over all participants revealed that a high cardiac interoceptive accuracy was positivly related to enhanced neuronal engagement in the bilateral central dorsal insula in response to olfactory stimulation (right: *T* = 3.71, FWE_corr_ ≤ .042; MNI: 45,2,−4; left: *T* = 3.51, FWE_corr_ ≤ .048; MNI: −45,4,−4). This observed neuronal engagement was located in those insular subdivisions reported as interoceptive processing areas in the meta‐analysis (Kurth et al., 2010, compare Figure 3).

**Figure 4:**
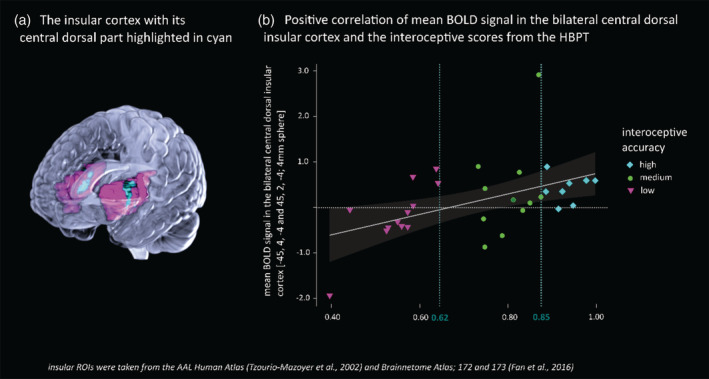
Mean BOLD signal changes in the bilateral central dorsal insula in relation to interoceptive accuracy. All participants with high interoceptive accuracy, but only 20% of those participants with low interoceptive accuracy, presented an increased BOLD signal in the insular ROI. These results remain significant (*p* = .032) after the exclusion of the two outliers (−1.93 and 2.91). The categorization in low/medium/high cardiac interoceptive accuracy is explained in detail in the statistical methods

All of those eight participants who showed a high cardiac interoceptive accuracy presented an increased BOLD signal in the bilateral central dorsal insular ROI. For the medium‐scoring individuals approximately two‐third had enhanced BOLD responses, however, in case of the low scorers only 20% presented a stronger BOLD Signal (Figure 4).

**Figure 5:**
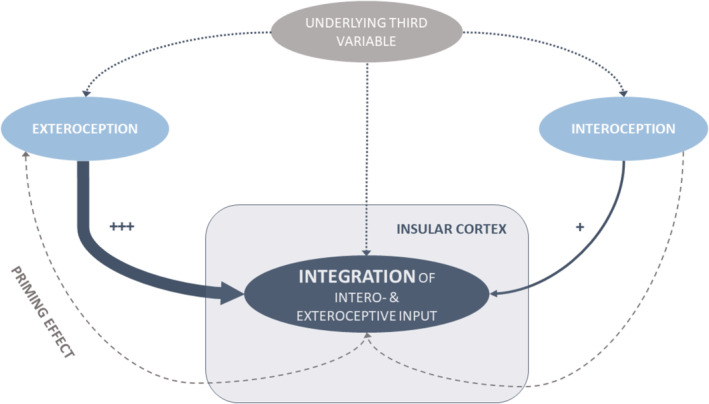
Overview of explanatory models describing the coherence of intero‐ and exteroception. Main explanatory model: External stimuli such as olfactory stimulation trigger (+++) the integration of both, extero‐ and interoceptive input, reflected in enhanced neuronal responses in the central dorsal insular cortex. Alternative model 1: Interoceptive sensations serve as priming factors for incoming external sensations. Alternative model 2: A common underlying variable such as situational attention influences both intero‐ and exteroception

## 4 DISCUSSION

In line with our hypothesis, we observed a positive relation between cardiac interoceptive accuracy and olfactory exteroceptive processing. This relation was reflected in both olfactory testing and dorsal insula reactivity during olfactory stimulation.

The mean cardiac interoceptive accuracy of 0.71–0.76 proved to be reliable and consistent. However, it was considerably lower than the mean cardiac interoceptive accuracy score of 0.92 reported by Stern et al., 2017. This difference is most likely explained by the fact that Stern and colleagues presented participants several selection options for heartbeat counts during the HBPT, while the current study used an uncued response paradigm (i.e., particpants were simply asked to estimate their heartbeat count). Consistent with this, previous studies also applying the HBPT mental tracking method showed similar results to our mean scores, reporting a mean of 0.76 among normosmics (Krajnik et al., 2015) or a mean of 0.65, with data ranging from 0.15 to 0.99 (Tan et al., 2018).

In terms of the psychophysiological measurements of olfactory function, we observed a differential effect. While olfactory discrimination and identification related positively to cardiac interoceptive accuracy, olfactory threshold related negatively. Further, results from the combined regression analysis revealed that only olfactory discrimination contributed significantly to the explained variance of cardiac interoceptive accuracy. Taken together, the findings suggest that olfactory discrimination (primarily), and olfactory‐identification (to a lesser extent) are facilitated by cardiac interoceptive accuracy. Before turning to our theoretical model, it is important to explain why a differential role of cardiac interoceptive accuracy may exist across the olfactory tasks. Olfactory threshold represents the ability to perceive odors at low concentrations and subsequently illustrates the peripheral functions of the olfactory epithelium to some degree. Olfactory discrimination and identification, however, require a much stronger cognitive integration and categorization of the environment. Similar to the feature of discrimination in interoceptive awareness (Khalsa et al., 2018), the presented olfactory stimuli need to be localized, compared and finally classified as olfactory stimuli. These latter abilities might be shaped by the momentary needs of the body, as sensed via interoceptive afferents. Related to our results, a coherence between smelling abilities and cardiac interoceptive accuracy became evident in a prior study, reporting reduced cardiac interoceptive accuracy among dysosmic patients (Krajnik et al., 2015). Taken together, the findings from the current study indicate that olfactory discrimination (primarily) and identification (to a lesser extent) are facilitated by cardiac interoceptive accuracy.

We propose a main explanatory model as well as two alternative models of how intero‐ and exteroceptive functioning are related and our present results may guide the interpretation.

In our ***main explanatory model***, olfactory stimuli trigger the integration of intero‐ and exteroceptive sensations in the insular cortex (Figure 5). Refering to the example from the Introduction: The warm smell of freshly cooked bread triggers the integration of interoceptive input. The categorization and the relevance of this incoming olfactory sensation will be interpreted based on the momentarily incoming interoceptive cues. The match between the current bodily state—detected by interoceptive needs (e.g., low blood sugar levels)—and the incoming exteroceptive stimuli will determine the classification of the bread odor as relevant or irrelevant, and consequently guide subsequent motivational behaviour. Here, both salience and interoceptive processing go hand in hand: Interoception is crucial to constantly monitor the bodily state, which in turn influences the perceived salience of exteroceptive stimuli. Supporting this assumption, we found intensified processing within the dorsal, interoceptive parts of the insula in response to olfactory stimulation among the participants with high cardiac interoceptive accuracy. As this insular integration is only present in the subgroup of individuals with at least moderate interoceptive function, we suspect that for people with low interoceptive function the integrational process is limited and therefore no significant results became evident in the covariate analysis.

Besides our present results, the sensory convergence model postulated by Avery et al. (2017), already hints at the confluence of extero‐ and interoceptive information in the central dorsal insula based on shared neuronal networks.

Alternatively, at least two more models need to be discussed.

**Alternative model 1:** Interoceptive sensations serve as a priming factor for exteroceptive stimuli. The kind of priming is dependant on the individual sensitivity to interoceptive sensations, such as low blood sugar levels. In that case, a person would sense low blood sugar levels and then orient towards the smell of freshly cooked bread. Hence, the olfactory stimuli would be perceived more easily by persons who are intensly primed by their interoceptive sensations. However, the interoceptive integration would not be trigggered by the odor itself, but be present independent of the current surrounding. In contrast to our main explanatory model the sudden presentation of an odor would not amplify the interoceptive sensation.

In our study, olfactory stimulation evoked enhanced activation in interoceptive areas of the insular cortex. This contradicts alternative model 1.

**Alternative model 2:** The relation of cardiac interoceptive accuracy and olfactory function is explained by a common underlying variable, such as situational attention. People with high situational attention may be more attentive to both, intero‐ as well as exteroceptive stimuli. As situational attention might differe across ages, this underlying variable could also explain why the duration of anosmia is negatively related to interoceptive abilities in the study of Krajnik et al. (2015). However, the ages of their participants showed a wide range from 22 to 73 with a mean age of around 54 years—therefore, it seems unlikely to attribute the described relation solely on the age‐dependent situational attention.

In our view, the main explanatory model fits best with the comprehensive functional integration of the insular structure. However, most likely all models represent part of the truth and can be integrated in the framework of predictive coding, as every perception process serves to update the a priori models for future perceptions (Barrett & Simmons, 2015; Kleckner et al., 2017; Paulus, Feinstein, & Khalsa, 2019). The enhanced activation in the bilateral insula during anticipation processing (Simmons et al., 2006) probably represents the ongoing generation of newly calculated prediction signals for the events to come.

Spinning the prediction‐model‐theory further, as Paulus and Stein (2010) propose, inflexible internal models in an ever‐changing environement might pave the way for mental disorders. This suggestion is supported by both structural and functional impairments of the insular cortex in depressed individuals (Rottstadt et al., 2018). Major depressive disorder is characterized by both, impaired interoceptive abilities (Wiebking et al., 2015) as well as exteroceptive abilities, especially by impaired olfactory function (Atanasova et al., 2008; Croy & Hummel, 2017). In line with these findings, we assume a disturbed insular integration processing in depression, which is also reflected in a misbalance of intero‐ and exteroception (Croy & Hummel, 2017; Khalsa et al., 2018; Paulus & Stein, 2010; Quadt, Critchley, & Garfinkel, 2018) and subsequently, impaired salience processing (Yang et al., 2016). Still, to our knowledge, there are no studies focusing on these variables in one clinical study sample.

If we correctly believe that our results demonstrate a relation between interoceptive and exteroceptive abilities, this relation may generalize to other exteroceptive modalities. Still, no coherence between interoceptive accuracy and visual exteroceptive skills were found in a study sample of healthy individuals (Stern et al., 2017). For gustation, however, Avery et al. (2017) introduced a sensory convergence model, describing a group of neurons in the dorsal mid‐insula being selectively sensitive to gustatory and interoceptive stimuli (Avery et al., 2017). Thus, olfaction may be more related to interoception (i.e., in comparision to the other exteroceptive senses), due to the strong and early insular involvement in olfactory processing. This insular involvement is reflected in the specialized chemosensory domain located in the central and anterior‐dorsal insular cortex (Kurth et al., 2010). Further, this shared chemosensory domain encompasses the two fundamental sensory channels for eating behaviour, the olfactory and gustatory system (Aschenbrenner et al., 2008; Hummel & Nordin, 2005; Kurth et al., 2010).

This is one of the very few studies focusing on olfactory function and cardiac interoceptive accuracy in one study sample. Still, the study has its limitations: *Different interoceptive measurements may show different relations to exteroceptive abilities*. The transferability of cardiac interoceptive accuracy to other modalities is shown to be limited: cardiac interoceptive accuracy is not significantly correlated to respiratory interoceptive accuracy (Garfinkel et al., 2016) and similar results have been reported for gastric perception, heartbeat perception, proprioception, pain, balance and bitter taste by Ferentzi et al. (2018). *Further, it remains unclear how the different features of interoceptive awareness transfer to each other*. By assessing the cardiac interoceptive accuracy, we do not know how these results transfer to other features of interoceptive awareness, such as the interoceptive sensibility and the insight. *Olfactory function was tested in an established two dimensional approach, while cardiac interoceptive accuracy was assessed by the restricted framework of the HBPT*. Although the HBPT has been used in various experimental set‐ups, for instance in (Krajnik et al., 2015; Stern et al., 2017), it is important to note that it can be biased by assumed hearbeat rates (Brener & Ring, 2016). *The objective measurements of the HBPT were performed by a clinical physician*. Although general practitioners are very used to counting pulses, this might have added a confound to the experimental set‐up. Nevertheless, we are positive that this potential confounder is minor, as the HBPT results in this study are comparable to results obtained by physiological measurements such as a puls oximeter in prior studies (Ferentzi, Horváth, & Köteles, 2019; Stevenson, Francis, Oaten, & Schilt, 2018; Tan et al., 2018).

We conclude that a multidimensional approach for future studies focusing on the interaction of intero‐ and exteroceptive abilities is required. This includes the testing of different intero‐ and exteroceptive modalities as well as the precise distinction of the different interoceptive features. To be able to further detangle interoceptive abilities from higher cognitive functions such as concentration capacity, general accuracy and sustained alertness, the collection of attentional data (e.g., by the application of a simple paper and pencil test, such as the d2‐test [Brickenkamp, Liepmann, & Schmidt‐Atzert, 2010]) is crucial.

## 5 CONCLUSION

The current study points out further indications on the relation of interoceptive and exteroceptive processing. We reported a positive correlation of olfactory discrimination as well as olfactory identification and mean cardiac interoceptive accuracy scores. We also described enhanced activation in the insula following olfactory stimulation among participants with strong cardiac interoceptive accuracy. This activation was found in dorsal insular subdivsions reported as functional domains of interoceptive processing. Based on the results, we discussed three explanatory approaches for the link between intero‐ and exteroceptive processing. In our view, olfactory stimulation serves as an exteroceptive trigger for interoceptive integration. Due to the limitations of the study, these findings are exploratory and need to be confirmed in future studies. The data, therefore, may serve as a first step and guide future research on the fundamental relation of exteroceptive chemosensory processing and interoceptive processing based on the common core structure, the central dorsal‐insula.

## CONFLICT OF INTEREST

The authors have no conflict of interest to declare.

## Supporting information


**Table S1** Olfactory activation cluster (p<.001 uncorr). FWE corrected small volume analysis for the pre‐defined regions of interest is reported in the main manuscript.

**Table S2** Covariate analysis activation cluster (p<.001 uncorr). FWE corrected small volume analysis for the predefined regions of interest is reported in the main manuscript.

## Data Availability

The data that support the findings of this study are available on request from the corresponding author. The data are not publicly available due to privacy or ethical restrictions.

## References

[hbm24985-bib-0001] Aschenbrenner, K. , Hummel, C. , Teszmer, K. , Krone, F. , Ishimaru, T. , Seo, H. S. , & Hummel, T. (2008). The influence of olfactory loss on dietary behaviors. Laryngoscope, 118(1), 135–144. 10.1097/MLG.0b013e318155a4b9 17975508

[hbm24985-bib-0002] Atanasova, B. , Graux, J. , El Hage, W. , Hommet, C. , Camus, V. , & Belzung, C. (2008). Olfaction: A potential cognitive marker of psychiatric disorders. Neuroscience and Biobehavioral Reviews, 32(7), 1315–1325. 10.1016/j.neubiorev.2008.05.003 18555528

[hbm24985-bib-0003] Avery, J. A. , Gotts, S. J. , Kerr, K. L. , Burrows, K. , Ingeholm, J. E. , Bodurka, J. , … Kyle Simmons, W. (2017). Convergent gustatory and viscerosensory processing in the human dorsal mid‐insula. Human Brain Mapping, 38(4), 2150–2164. 10.1002/hbm.23510 28070928 PMC5575915

[hbm24985-bib-0004] Barrett, L. F. , & Simmons, W. K. (2015). Interoceptive predictions in the brain. Nature Reviews. Neuroscience, 16(7), 419–429. 10.1038/nrn3950 26016744 PMC4731102

[hbm24985-bib-0005] Brener, J. , & Ring, C. (2016). Towards a psychophysics of interoceptive processes: The measurement of heartbeat detection. Philosophical Transactions of the Royal Society of London. Series B, Biological Sciences, 371(1708), 20160015 10.1098/rstb.2016.0015 28080972 PMC5062103

[hbm24985-bib-0006] Brickenkamp, R. , Liepmann, D. , & Schmidt‐Atzert, L. (2010). Test d2—Revision: Aufmerksamkeits‐ und Konzentrationstest. Göttingen, Germany: Hogrefe.

[hbm24985-bib-0007] Craig, A. D. (2002). How do you feel? Interoception: The sense of the physiological condition of the body. Nature Reviews. Neuroscience, 3(8), 655–666. 10.1038/nrn894 12154366

[hbm24985-bib-0008] Craig, A. D. (2007). Interoception and emotion: A neuroanatomical perspective In Lewis M., Haviland‐Jones J. M., & Barrett L. F. (Eds.), Handbook of emotion, chapter 16 (pp. 272–288). New York: Guilford Press.

[hbm24985-bib-0009] Craig, A. D. (2009). How do you feel—now? The anterior insula and human awareness. Nature Reviews. Neuroscience, 10(1), 59–70. 10.1038/nrn2555 19096369

[hbm24985-bib-0010] Craig, A. D. (2011). Significance of the insula for the evolution of human awareness of feelings from the body. Annals of the new York Academy of Sciences, 1225, 72–82. 10.1111/j.1749-6632.2011.05990.x 21534994

[hbm24985-bib-0011] Croy, I. , & Hummel, T. (2017). Olfaction as a marker for depression. Journal of Neurology, 264(4), 631–638. 10.1007/s00415-016-8227-8 27393116

[hbm24985-bib-0012] Damasio, A. (1999). The feeling of what happens: Body and emotion in the making of consciousness. Fort Worth,TX: Harcourt College Publishers.

[hbm24985-bib-0013] Fan, L. , Li, H. , Zhuo, J. , Zhang, Y. , Wang, J. , Chen, L. , … Jiang, T. (2016). The human Brainnetome atlas: A new brain atlas based on connectional architecture. Cerebral Cortex, 26(8), 3508–3526. 10.1093/cercor/bhw157 27230218 PMC4961028

[hbm24985-bib-0014] Ferentzi, E. , Bogdany, T. , Szabolcs, Z. , Csala, B. , Horvath, A. , & Koteles, F. (2018). Multichannel investigation of interoception: Sensitivity is not a generalizable feature. Frontiers in Human Neuroscience, 12, 223 10.3389/fnhum.2018.00223 29910718 PMC5992275

[hbm24985-bib-0015] Ferentzi, E. , Horváth, Á. , & Köteles, F. (2019). Do body‐related sensations make feel us better? Subjective well‐being is associated only with the subjective aspect of interoception. Psychophysiology, 56(4), e13319 10.1111/psyp.13319 30629298

[hbm24985-bib-0016] Garfinkel, S. N. , Manassei, M. F. , Hamilton‐Fletcher, G. , In den Bosch, Y. , Critchley, H. D. , & Engels, M. (2016). Interoceptive dimensions across cardiac and respiratory axes. Philosophical Transactions of the Royal Society of London. Series B, Biological Sciences, 371(1708), 20160014 10.1098/rstb.2016.0014 28080971 PMC5062102

[hbm24985-bib-0017] Garfinkel, S. N. , Seth, A. K. , Barrett, A. B. , Suzuki, K. , & Critchley, H. D. (2015). Knowing your own heart: Distinguishing interoceptive accuracy from interoceptive awareness. Biological Psychology, 104, 65–74. 10.1016/j.biopsycho.2014.11.004 25451381

[hbm24985-bib-0018] Ghaziri, J. , Tucholka, A. , Girard, G. , Boucher, O. , Houde, J. C. , Descoteaux, M. , … Nguyen, D. K. (2018). Subcortical structural connectivity of insular subregions. Scientific Reports, 8(1), 8596 10.1038/s41598-018-26995-0 29872212 PMC5988839

[hbm24985-bib-0019] Ghaziri, J. , Tucholka, A. , Girard, G. , Houde, J. C. , Boucher, O. , Gilbert, G. , … Nguyen, D. K. (2017). The Corticocortical structural connectivity of the human insula. Cerebral Cortex, 27(2), 1216–1228. 10.1093/cercor/bhv308 26683170

[hbm24985-bib-0020] Gottfried, J. A. (2006). Smell: Central nervous processing. Advances in Oto‐Rhino‐Laryngology, 63, 44–69. 10.1159/000093750 16733332

[hbm24985-bib-0021] Hedner, M. , Larsson, M. , Arnold, N. , Zucco, G. M. , & Hummel, T. (2010). Cognitive factors in odor detection, odor discrimination, and odor identification tasks. Journal of Clinical and Experimental Neuropsychology, 32(10), 1062–1067. 10.1080/13803391003683070 20437286

[hbm24985-bib-0022] Hummel, T. , Kobal, G. , Gudziol, H. , & Mackay‐Sim, A. (2007). Normative data for the "Sniffin' Sticks" including tests of odor identification, odor discrimination, and olfactory thresholds: An upgrade based on a group of more than 3,000 subjects. European Archives of Oto‐Rhino‐Laryngology, 264(3), 237–243. 10.1007/s00405-006-0173-0 17021776

[hbm24985-bib-0023] Hummel, T. , & Nordin, S. (2005). Olfactory disorders and their consequences for quality of life. Acta Oto‐Laryngologica, 125(2), 116–121. 10.1080/00016480410022787 15880938

[hbm24985-bib-0024] Kelly, C. , Toro, R. , Di Martino, A. , Cox, C. L. , Bellec, P. , Castellanos, F. X. , & Milham, M. P. (2012). A convergent functional architecture of the insula emerges across imaging modalities. NeuroImage, 61(4), 1129–1142. 10.1016/j.neuroimage.2012.03.021 22440648 PMC3376229

[hbm24985-bib-0025] Khalsa, S. S. , Adolphs, R. , Cameron, O. G. , Critchley, H. D. , Davenport, P. W. , Feinstein, J. S. , … Zucker, N. (2018). Interoception and mental health: A roadmap. Biological Psychiatry: Cognitive Neuroscience and Neuroimaging, 3(6), 501–513. 10.1016/j.bpsc.2017.12.004 29884281 PMC6054486

[hbm24985-bib-0026] Kleckner, I. R. , Zhang, J. , Touroutoglou, A. , Chanes, L. , Xia, C. , Simmons, W. K. , … Barrett, L. F. (2017). Evidence for a large‐scale brain system supporting allostasis and interoception in humans. Nature Human Behaviour, 1, 0069 10.1038/s41562-017-0069 PMC5624222 28983518

[hbm24985-bib-0027] Krajnik, J. , Kollndorfer, K. , Notter, L. A. , Mueller, C. A. , & Schopf, V. (2015). The impact of olfactory dysfunction on interoceptive awareness. Psychophysiology, 52(2), 263–268. 10.1111/psyp.12316 25109393 PMC4594750

[hbm24985-bib-0028] Kroenke, K. , Spitzer, R. L. , Williams, J. B. , & Lowe, B. (2010). The patient health questionnaire somatic, anxiety, and depressive symptom scales: A systematic review. General Hospital Psychiatry, 32(4), 345–359. 10.1016/j.genhosppsych.2010.03.006 20633738

[hbm24985-bib-0029] Kurth, F. , Zilles, K. , Fox, P. T. , Laird, A. R. , & Eickhoff, S. B. (2010). A link between the systems: Functional differentiation and integration within the human insula revealed by meta‐analysis. Brain Structure & Function, 214(5–6), 519–534. 10.1007/s00429-010-0255-z 20512376 PMC4801482

[hbm24985-bib-0030] Lambert, C. , Simon, H. , Colman, J. , & Barrick, T. R. (2017). Defining thalamic nuclei and topographic connectivity gradients in vivo. NeuroImage, 158, 466–479. 10.1016/j.neuroimage.2016.08.028 27639355

[hbm24985-bib-0031] Maldjian, J. A. , Laurienti, P. J. , Kraft, R. A. , & Burdette, J. H. (2003). An automated method for neuroanatomic and cytoarchitectonic atlas‐based interrogation of fMRI data sets. Neuroimage, 19(3), 1233–1239. Retrieved from. https://www.ncbi.nlm.nih.gov/pubmed/12880848 12880848 10.1016/s1053-8119(03)00169-1

[hbm24985-bib-0032] Matthew Brett, J.‐L. A. , Valabregue, R. , Poline, J.‐B . (2002). *Region of interest analysis using an SPM toolbox [abstract]*. Paper presented at the presented at the 8th International Conference on Functional Mapping of the Human Brain, June 2–6, Sendai, Japan.

[hbm24985-bib-0033] Mazzola, L. , Royet, J. P. , Catenoix, H. , Montavont, A. , Isnard, J. , & Mauguiere, F. (2017). Gustatory and olfactory responses to stimulation of the human insula. Annals of Neurology, 82(3), 360–370. 10.1002/ana.25010 28796326

[hbm24985-bib-0034] Menon, V. , & Uddin, L. Q. (2010). Saliency, switching, attention and control: A network model of insula function. Brain Structure & Function, 214(5–6), 655–667. 10.1007/s00429-010-0262-0 20512370 PMC2899886

[hbm24985-bib-0035] Paulus, M. P. , Feinstein, J. S. , & Khalsa, S. S. (2019). An active inference approach to interoceptive psychopathology. Annual Review of Clinical Psychology, 15, 97–122. 10.1146/annurev-clinpsy-050718-095617 PMC7280559 31067416

[hbm24985-bib-0036] Paulus, M. P. , & Stein, M. B. (2010). Interoception in anxiety and depression. Brain Structure & Function, 214(5–6), 451–463. 10.1007/s00429-010-0258-9 20490545 PMC2886901

[hbm24985-bib-0037] Pollatos, O. , Gramann, K. , & Schandry, R. (2007). Neural systems connecting interoceptive awareness and feelings. Human Brain Mapping, 28(1), 9–18. 10.1002/hbm.20258 16729289 PMC6871500

[hbm24985-bib-0038] Quadt, L. , Critchley, H. D. , & Garfinkel, S. N. (2018). The neurobiology of interoception in health and disease. Annals of the New York Academy of Sciences, 1428(1), 112–128. 10.1111/nyas.13915 29974959

[hbm24985-bib-0039] Rottstadt, F. , Han, P. , Weidner, K. , Schellong, J. , Wolff‐Stephan, S. , Strauss, T. , … Croy, I. (2018). Reduced olfactory bulb volume in depression—A structural moderator analysis. Human Brain Mapping, 39, 2573–2582. 10.1002/hbm.24024 29493048 PMC6866619

[hbm24985-bib-0040] Schandry, R. (1981). Heart beat perception and emotional experience. Psychophysiology, 18(4), 483–488. Retrieved from. https://www.ncbi.nlm.nih.gov/pubmed/7267933 7267933 10.1111/j.1469-8986.1981.tb02486.x

[hbm24985-bib-0041] Sezille, C. , Messaoudi, B. , Bertrand, A. , Joussain, P. , Thevenet, M. , & Bensafi, M. (2013). A portable experimental apparatus for human olfactory fMRI experiments. Journal of Neuroscience Methods, 218(1), 29–38. 10.1016/j.jneumeth.2013.04.021 23660526

[hbm24985-bib-0099] Simmons, A. , Strigo, I. , Matthews, S. C. , Paulus, M. P. , & Stein, M. B. (2006). Anticipation of aversive visual stimuli is associated with increased insula activation in anxiety‐prone subjects. Biol Psychiatry, 60(4), 402–409. 10.1016/j.biopsych.2006.04.038 16919527

[hbm24985-bib-0042] Stern, E. R. , Grimaldi, S. J. , Muratore, A. , Murrough, J. , Leibu, E. , Fleysher, L. , … Burdick, K. E. (2017). Neural correlates of interoception: Effects of interoceptive focus and relationship to dimensional measures of body awareness. Human Brain Mapping, 38(12), 6068–6082. 10.1002/hbm.23811 28901713 PMC5757871

[hbm24985-bib-0043] Stevenson, R. J. , Francis, H. M. , Oaten, M. J. , & Schilt, R. (2018). Hippocampal dependent neuropsychological tests and their relationship to measures of cardiac and self‐report interoception. Brain and Cognition, 123, 23–29. 10.1016/j.bandc.2018.02.008 29505942

[hbm24985-bib-0044] Tan, Y. , Wei, D. , Zhang, M. , Yang, J. , Jelincic, V. , & Qiu, J. (2018). The role of mid‐insula in the relationship between cardiac interoceptive attention and anxiety: Evidence from an fMRI study. Scientific Reports, 8(1), 17280 10.1038/s41598-018-35635-6 30467392 PMC6250688

[hbm24985-bib-0045] Uddin, L. Q. (2015). Salience processing and insular cortical function and dysfunction. Nature Reviews. Neuroscience, 16(1), 55–61. 10.1038/nrn3857 25406711

[hbm24985-bib-0046] van der Kooy, D. , Koda, L. Y. , McGinty, J. F. , Gerfen, C. R. , & Bloom, F. E. (1984). The organization of projections from the cortex, amygdala, and hypothalamus to the nucleus of the solitary tract in rat. The Journal of Comparative Neurology, 224(1), 1–24. 10.1002/cne.902240102 6715573

[hbm24985-bib-0047] Wiebking, C. , de Greck, M. , Duncan, N. W. , Tempelmann, C. , Bajbouj, M. , & Northoff, G. (2015). Interoception in insula subregions as a possible state marker for depression‐an exploratory fMRI study investigating healthy, depressed and remitted participants. Frontiers in Behavioral Neuroscience, 9, 82 10.3389/fnbeh.2015.00082 25914633 PMC4392695

[hbm24985-bib-0048] Yang, Y. , Zhong, N. , Imamura, K. , Lu, S. , Li, M. , Zhou, H. , … Li, K. (2016). Task and resting‐state fMRI reveal altered salience responses to positive stimuli in patients with major depressive disorder. PLoS One, 11(5), e0155092 10.1371/journal.pone.0155092 27192082 PMC4871416

