# Supplementary figures and images for: Interoceptive accuracy and its impact on neuronal responses to olfactory stimulation in the insular cortex
Source: Hum Brain Mapp. 2020 Mar 26;41(11):2898–908. doi: 10.1002/hbm.24985 (PMC7336161; doi:10.1002/hbm.24985)

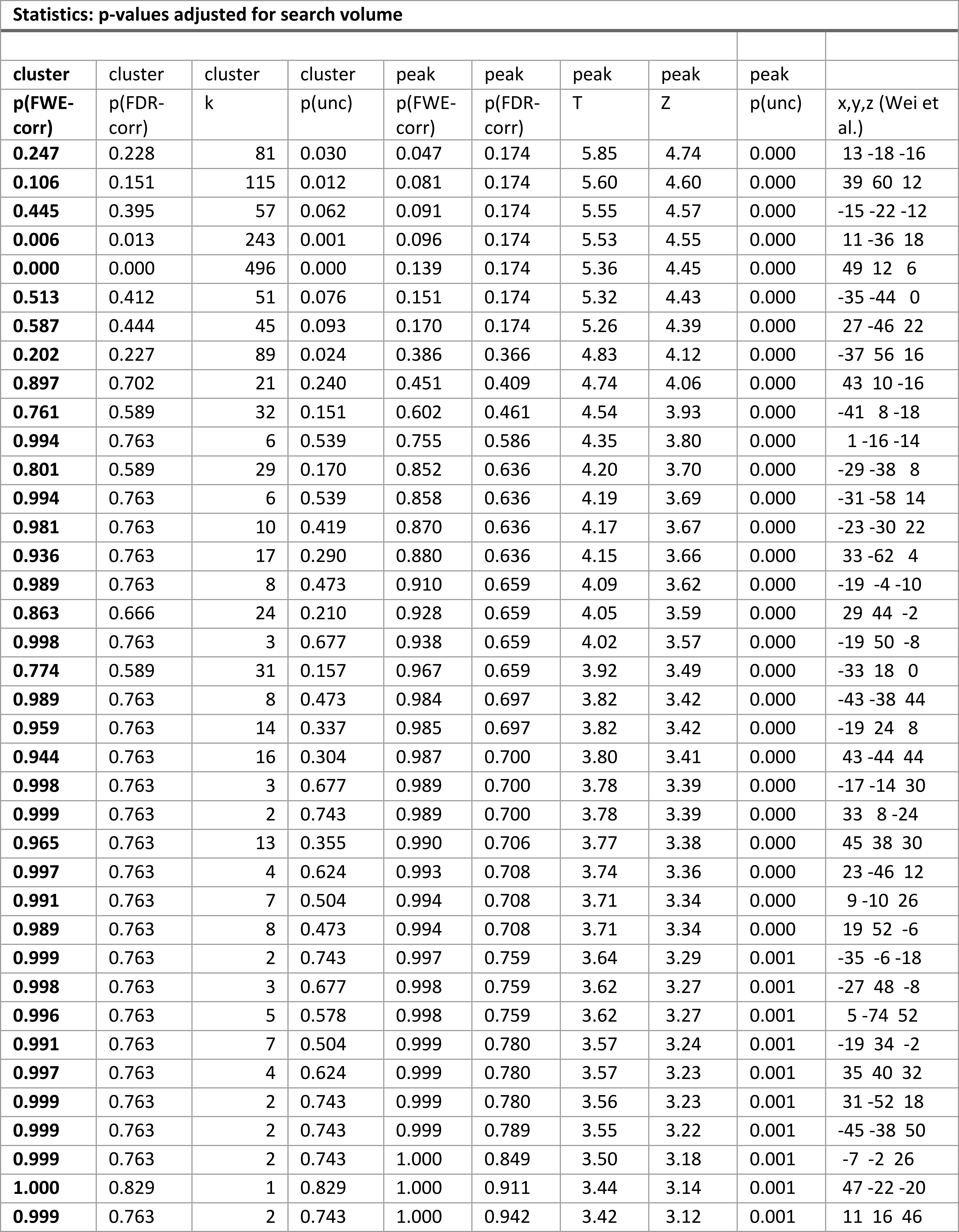

Supplement: Supplementary file 1 — Table S1 Olfactory activation cluster (p<.001 uncorr). FWE corrected small volume analysis for the pre‐defined regions of interest is reported in the main manuscript. [file HBM-41-2898-s001.png]

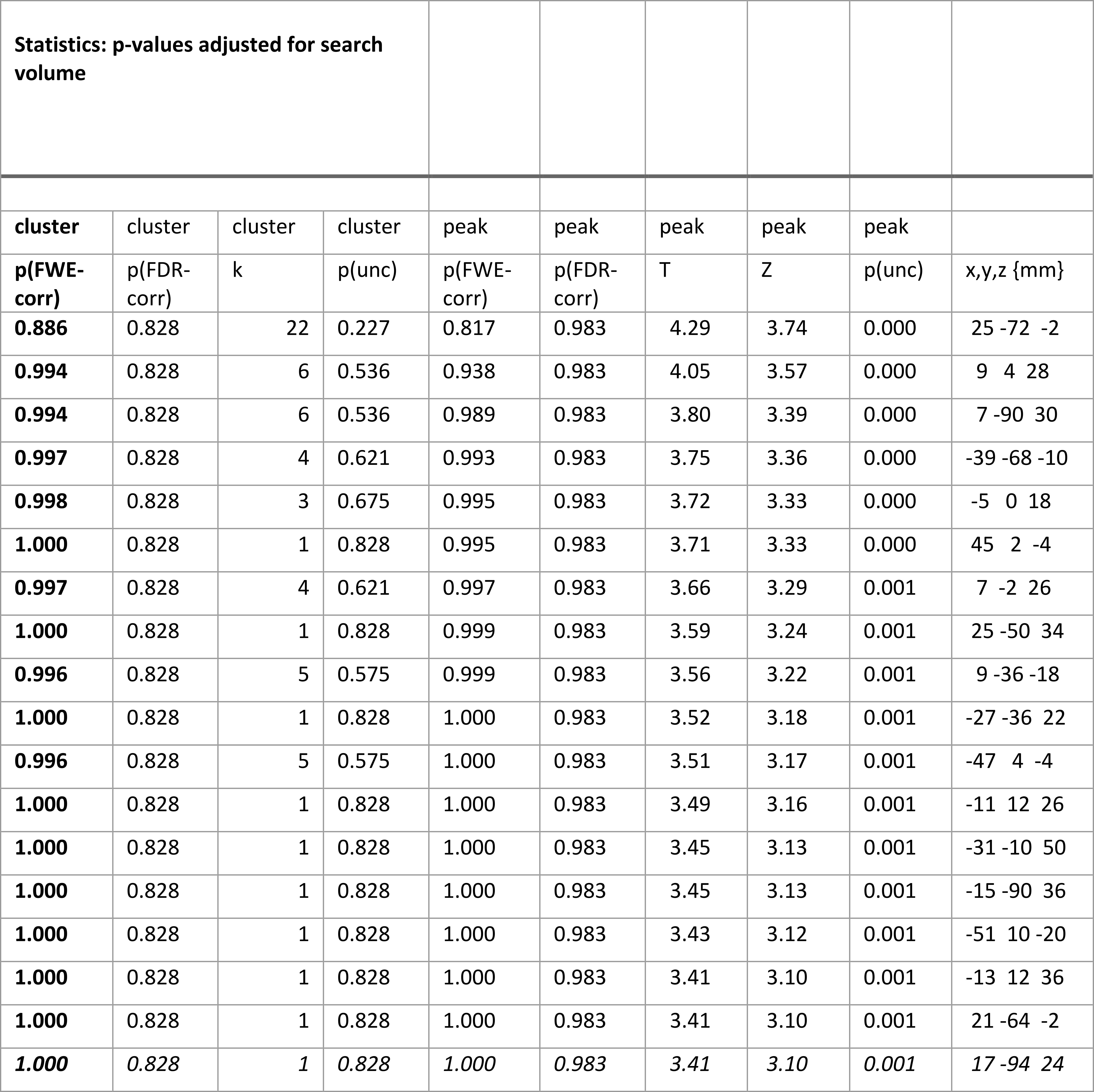

Supplement: Supplementary file 2 — Table S2 Covariate analysis activation cluster (p<.001 uncorr). FWE corrected small volume analysis for the predefined regions of interest is reported in the main manuscript. [file HBM-41-2898-s002.png]
